# Dr. Lloyd’s Loss by Fire

**Published:** 1891-03

**Authors:** 


DR. L. LLOYD, the distinguished representative in the lower house of the present Texas Legislature, had the misfortune to lose his elegant new home, in Jacksonville, early in this month. He had just finished it, and had made every arrangement for spending his life comfortably; had surrounded his family with all the comforts of an elegant modern home; had built capacious barns, and all the necessary out-houses, and had harvested his crops of corn, oats, fodder, potatoes, and other farm products usualy stored by the thrifty husbandman; had his carriage house and stables, and all just completed, when the destructive fiend swept it all away in a brief hour. The loss is the more sad from the fact that there was not a dollar of insurance on the property, and he lost all—house and contents, out-houses and grain, library, instruments, and all. His policy of insurance expired just after he left home for Austin, and he neglected to renew it, thinking he would do so on his first trip home; but alas, it was delayed too long. So many instances have occurred of a fire just after lapse of policy, that it looks as if there were a kind of fiendish intelligence that watches for and takes advantage of such lapses; the interval of time between lapse and renewal seems to be the danger period. It is safest to never allow a lapse. The good doctor has the sincere sympathy of the JOURNAL, of which he has been a faithful and long-time supporter; and of a host of attached friends.

